# A simulation training of family management for parents of children with epilepsy: a randomized clinical trial

**DOI:** 10.1186/s13052-024-01646-5

**Published:** 2024-04-19

**Authors:** Hua-yan Liu, Shan Zeng, Yue-wei Chen, Min Yi, Xiao-yan Tan, Jian-hui Xie, Xia Wu, Li-hui Zhu

**Affiliations:** 1 Hunan Children’s Hospital, Changsha, Hunan China; 2 Hunan University of Chinese Medicine, Changsha, Hunan China

**Keywords:** Epilepsy, Family management, Children, Simulation training

## Abstract

**Background:**

Epilepsy is a chronic neurological disorder that is more likely to be diagnosed in children. The main treatment involves long-term use of anti-epileptic drugs and above all, home care is of great importance. As there has not been a widely accepted home care protocols, simulating a home care environment is necessary for caregivers to develop skills of proper home care. This study aims to evaluate the effectiveness of a simulation training of family management style (STOFMS) for parents of children with epilepsy in China.

**Methods:**

A randomized controlled trial was conducted on 463 children with epilepsy and their families. They were recruited from March 2020 to November 2022 and randomly assigned to the STOFMS group or the conventional group in a 1:1 ratio. Scores of family management measures, 8-item of Morisky Medication Adherence and epilepsy clinical symptom of both groups were collected at three points of time: within 24 h after admission (T0), 3 months after discharge (T1), and 6 months after discharge (T2). Changes due to intervention were compared across groups by repeated-measures ANOVA. The study report followed the CONSORT 2010 checklist.

**Results:**

There were statistically significant differences between the two groups at T2. A considerable increase over the baseline was observed in the total management level score and subscale scores in the STOFMS group at T1, compared with essentially no change in the control group. In terms of medication adherence, the STOFMS group performance improved greatly at T1 and T2 compared with the control group. The same result was also found in clinical efficacy at T2 (*p* < 0.05).

**Conclusion:**

STOFMS is an effective intervention to improve family management level, treatment adherence and clinical efficacy for children with epilepsy.

**Trial registration:**

The registration number is ChiCTR2200065128. Registered at 18 October 2022, http://www.medresman.org.cn

## Introduction

Epilepsy is a common chronic neurological disorder characterized by highly synchronized and abnormal neuronal activity in the brain. It can cause unpredictable and recurrent seizures [1]. According to a global epidemiological survey, an estimated 50 to 70 million people worldwide are living with epilepsy, approximately 80% of whom live in developing countries, accounting for 0.6% of the total global disease burden [2, 3]. The Global Health Statistics report stated that the number of patients diagnosed with epilepsy in China has increased by 259% over 25 years, from 2.63 million in 1990 to 9.84 million in 2015 [4]. Nearly 41% of patients with epilepsy in China have a treatment gap, as reported by the Global Campaign Against Epilepsy [5]. Epilepsy is observed across all age groups, with a higher prevalence in male children [6]. Seizures can significantly disrupt children’s development of intellectual, cognitive, and memory functioning [7].

At present, the treatment of childhood epilepsy is still a challenging issue [8]. So far, the recognized effective treatment is the rational, regular, and long-term use of anti-epileptic drugs [9]. However, approximately one-third of newly diagnosed children with epilepsy cannot benefit from this treatment due to poor medication adherence [10]. Despite interventions aiming to promote treatment adherence, such as health education about epilepsy and consequences of non-adherence, as well as programme to enhance problem-solving skills for families with children with epilepsy [11, 12], the medication adherence is subject to children’s self-regulatory capacity and their need for full-time parental care. Therefore, it is crucial to pay special attention to children’s family management levels.

The Family Management Style Framework (FMSF) refers to the entire family working as a team to organize, integrate, and accomplish the tasks associated to condition management of children with chronic illnesses [13]. Current evidence has indicated that FMSF can improve the treatment adherence and health outcomes in pediatric rheumatism and asthma [14, 15]. It is also conducive to children’s disease prevention, medication adherence and the quality of life [16, 17].

Previous studies showed that the family management plan can facilitate family’s ability of managing children with epilepsy. As family management is a long-term process, parents are advised to follow the family management plan to offer home care after discharge. However, family management may not show good outcomes for those fail to fully comply with the plan since researchers cannot monitor families’ behaviors and skills all the time [18]. Meanwhile, poor medication adherence was identified as a major cause in follow-up surveys, since many parents did not strictly implement the suggested family management plan after discharge. Researchers in this study designed an exploratory improvement programme based on preliminary work which simulated post-discharge family management during the hospitalization period. In the programme, parents’ family management ability was assessed the day before the child was discharged. Parents who failed the assessment would continue to receive simulation training and assessment again on the day of discharge. If they failed the second assessment, the medical personnel would offer post-discharge guidance through home visit. The simulation training of family management style (STOFMS) is a flexible and family-centered intervention that focuses on improving caregivers’ perceptions of disease and problem-solving capability [13]. It teaches the family members how to manage both the child’s chronic medical conditions and daily living activities. Previous literature has proved the effectiveness of this strategy in achieving better treatment adherence and health outcomes of pediatric patients with rheumatism and asthma [14, 15]. As far as the researchers know, STOFMS has not been applied in children with epilepsy, for whom caregivers’ supervision is essential to disease management. Therefore, simulation training was included during the STOFMS group’s hospitalization. Ongoing support was given via the Internet to ensure that caregivers in both groups maintained control of the model and apply what they learned before discharge. The researchers applied this new method to children with epilepsy to enhance family management measures, medication adherence, and alleviate the clinical symptoms of epilepsy.

The researchers conducted a randomized controlled trial (RCT) in this study to investigate the efficacy of STOFMS on the family management level as compared with that of routine care, and medication adherence as well as clinical efficacy for children with epilepsy.

## Materials and methods

### Study design

The researchers conducted a single-blinded, randomized controlled trial in children with epilepsy in one tertiary hospital of China. Participants were recruited from March 2020 to November 2022. The study design, implementation and analysis followed the CONSORT statement.

### Population and participants

The inclusion criteria for children were (1) aged over 28 days to 14 years; (2) diagnosed with epilepsy, whereas the exclusion criteria were children with status epilepticus, epileptic encephalopathy, or other serious systemic diseases.

For the parents, the inclusion criteria were (1) with clear consciousness, able to express their wishes correctly; (2) willing to participate in this study and gave informed consent. Parents who (1) were not able to write, such as those with hearing or visual impairment; (2) experienced severe psychological trauma in the past 2 years; (3) suffered from serious organic or mental diseases such as heart, liver and kidney diseases were excluded from this study.

### Sample size

The sample size was calculated using PASS 15.0 software. By referring to the literature [19], the estimated score of the subscale of family management ability was ${\text{u}}1=38.28,\mathrm{ u}2=37.18,\upsigma =3.20$. By using the two-sided test, α = 0.05, 1-β = 0.90, the rate of loss to follow-up was 20%, and a total sample size of 448 cases was finally confirmed. 463 children with epilepsy and their parents were eventually included in the study.

### Procedures

#### Overall study design

Participants in the STOFMS group received STOFMS training, whereas those in the control group received Family Management Style Framework (FMSF) in the same period. We confirmed there were no significant intergroup differences in seizure frequency in the last 3 months and duration of each seizure. Physicians, nurses, and therapists assigned for both groups had the same medical qualifications. Assessments were conducted on the two groups at three time points: within 24 h after admission(T0), 3 months after discharge (T1), and 6 months after discharge (T2).

FMSF is an approach designed by a group of neurology experts based on the Framework for Family Management Measure created by Knafl et al. [20]. The intervention approach for the control group is detailed in the literature [18, 19].

#### Assessment and randomization method

##### Assessment

Children with epilepsy and their parents who both met the inclusion criteria were enrolled in the study by the admission nurses. Data collection and statistical analysis investigators who received special training from the research group were responsible for distributing and collecting the questionnaires which included the following information: the child’s age, gender and age of onset; parents’ age, gender, employment, and education; time since diagnosis, duration of medication, seizure frequency in the last 3 months, seizure duration, frequency of hospitalization in the past month, payment method and annual income, as well as Family Management Measure scale, which was used to assess how families managed children with chronic conditions.

##### Randomization method

This study was performed according to Consolidated Standards of Reporting Trials 2010 (CONSORT 2010) guidelines [21]. Administration nurses who were not involved in the intervention or data collection randomly assigned eligible individuals into the STOFMS or control group in a 1:1 ratio using the Random Allocation Software (version 1.0.0). To guarantee allocation concealment, sequentially numbered, opaque, sealed envelopes were prepared by the administration nurse. Participants of the two groups were allocated into different wards in the neurology department during the trial to avoid bias. Data collectors and analysts were blinded to the group allocation. The follow-up and data collection at each stage were completed by graduate students who did not participate in the study intervention or were aware of the grouping.

#### Intervention protocols

##### Control group

The control group was conducted with the FMSF [18, 19]. The specific implementation was as follows:

A. During hospitalization: (1) Form a multidisciplinary team of physicians, pharmacists and nurses to fully assess the child’s condition and jointly develop a family management plan. Next, investigate the use of anti-seizure medicine within 24 h of admission, and determine the long-term continuous treatment and medication plan based on the family’s economic status; (2) Set up family tasks, division of labor, and family support system; (3) Provide health education and establish personal electronic medical records; The Family Management Manual for Children with Epilepsy formulated by the neurology department was delivered to the children and their parents, and health education including personal guidance on disease knowledge and daily life was provided to them by the manual; 4) Offer medication guide on anti-seizure drugs, set an alarm clock to take medication, as well as deliver instructions on early identification and emergency treatment of seizures or aggravating symptoms.
B. Family self-management after discharge: (1) Manage the family environment. For example, adjust the lighting and avoid unnecessary sound stimulation to reduce children’s seizures; 2) Deliver personalized follow-up, monitor FMSF application and effect at home by phone calls, emails or Wechat every two weeks after discharge.

##### STOFMS group

Family management intervention was carried out in the STOFMS group during hospitalization, and simulation training of family management was added three days after admission. Follow-up was continued for 6 months after discharge to ensure that children and their parents were managing their life properly. The intervention was conducted as follows:

- Session 1-within 3 days after admission: (1) Identifying patient’s medical condition by the physician, pharmacist, nurse and family members; (2) Evaluating family’s ability to learn and afford anti-seizure drugs; (3) Determining the long-term treatment and medication regimen.
- Session 2–3 to 7 days after admission: (1) Establishing personal electronic files and educating participants how to use these records; (2) Providing proper instructions for taking anti-seizure drugs; (3) Educating family members on epilepsy prevention, early identification, emergency response and treatment of seizures or aggravating symptoms; (4) Assisting the whole family in assigning the household tasks, adjusting the family environment, and arranging activities including diet, nutrition, exercise, rest and sleep.
- Session 3–1 week after admission: (1) Reviewing the treatment plan in the *Family Management Manual for Children with Epilepsy*; (2) Asking the family members to identify their challenges in treatment adherence over the past week and setting corresponding treatment goals, problem-solving practices and detailed plans; (3) Identifying specific treatment adherence goals, problem-solving practices, and detailed plans; and (4) Simulating family management by setting up a family ward and guiding the children and their parents to view the ward as a family space and the doctors and nurses as family members. The simulation would last for 3 to 5 days, during which the doctors and nurses would observe the simulation process, record the problems, and correct them over time. The researchers would evaluate parents’ family management ability one day before discharge through theoretical examination and operational assessment, with a score of 80 or above considered qualified. If the children’s parents failed the assessment, they would continue to carry out family simulation training and be assessed again on the day of discharge. The child would not be discharged on time if their parents failed the second time, and the medical personnel would offer guidance at their homes after discharge. Our study showed that there was no family that didn't pass the second assessment.
- Sessions 4–3 to 6 months follow-up after discharge: conducting follow-up visits to monitor the efficacy of STOFMS every two weeks by telephone or Internet, and provide guidance and assistance to the children’s caregivers when needed.

#### Post-intervention and follow-up visits

Parents would download the Internet-based application developed by Hunan Children’s hospital, where children and their parents could get access to all kinds of information and health education materials, receive reminders for taking medicine and follow-up, and receive adherence questionnaires. For each follow-up visit, the medical personnel could check the relevant medical records in the system and extract follow-up information. Only authorized persons had access to the system information. In this study, all participants completed questionnaires, and data were collected before the intervention (T0), at 3-month follow-up (T1), and 6-month follow-up (T2).

#### Measures

##### Primary outcomes

1. Family Management Measure
  Family Management Measure (FaMM), first designed by Knafl et al. [20]., was used to assess how families managed caring for the children with chronic conditions. This study adopted the Chinese version of FaMM translated and revised by Zhang Ying et al. [22]. The scale has 53 items (including 17 reverse scoring items) across 6 factors: Child’s Daily Life, Condition Management Ability, Condition Management Effort, Family Life Difficulty, View of Condition Impact, and Parental Mutuality. The total score is 265, with items scored from 1 to 5, meaning completely disagree to completely agree. The Cronbach’s alpha value for the scales is 0.84 in China. In short, higher scores of Child’s Daily Life, Condition Management Ability, and Parental Mutuality indicate more ease in managing the child’s condition, whereas higher scores of Condition Management Effort, View of Condition Impact and Family Life Difficulty suggest more challenges in condition management.
2. 8-items Morisky Medication Adherence Scale
  The 8-item Morisky Medication Adherence Scale (MMAS-8) was developed by ISLAM T et al. [23] in 2008 to assess patients with hypertension in outpatient clinics. The Cronbach’s α coefficient of the source scale was 0.830. The scale includes 8 items with the first 7 items presented with Yes and No options. Each “No” response is rated as 1 and “Yes” as 0, except for item 5, which is in reverse. Item 8 uses a five-point Likert scale: never, occasionally, sometimes, frequently, and all time, correspondingly scored as of 1, 0.75, 0.50, 0.25, and 0 points. The total scale has a score range of 0–8, and the level of medication adherence is classified as low adherence (< 6), medium adherence (6–7), and high adherence (8).

##### Secondary outcomes

**Clinical efficacy**

The researchers recorded the number of seizures in each period, the duration of each seizure, and EEG results to judge children’s clinical efficacy. The criteria of different outcomes are described below [24].

Markedly effective: EEG returned to normal after treatment, clinical symptoms were completely relieved, and the frequency of seizures was reduced by more than 70%.

Effective: EEG was generally normal, clinical symptoms were significantly improved, and the frequency of seizures was reduced by 50%.

Ineffective: EEG and clinical symptoms were not significantly improved after treatment, and the frequency of seizures was reduced by 50% or less or there was no change. The number of effective cases was made up of remarkably effective and effective cases.

### Statistical analysis

Data collected at T0, T1, and T2 were analyzed using IBM SPSS™ Statistics, version 20.0 (IBM Corp.). Descriptive statistics, including frequency, percentage, mean and standard deviation, were employed to summarize baseline data and analyze the distribution of responses. The Chi-squared test was performed to compare the differences in basic characteristics between the STOFMS and control groups. Repeated-measures analysis of variance was used to compare children’s FaMM scores in different periods. A *p*-value < 0.05 was considered statistically significant.

## Results

### Participants

A total of 463 patients met the inclusion criteria (232 in the control group and 231 in the STOFMS group). In the control group, there were 33 families lost to follow-up, including 16 families not completing the information and 17 discontinuing intervention or follow-up at T1, 8 families dropped out at T2. Whereas in the STOFMS group, 25 families were lost to follow-up at T1, including 17 with incomplete information and 8 discontinuing intervention. And at T2, 11 families were lost to follow-up, in which 9 had incomplete information and 2 discontinued intervention. Moreover, 2 families were excluded from the STOFMS group due to invalid questionnaires during data analysis. Therefore, a total of 384 children with epilepsy and their parents (191 in the control group and 193 in the STOFMS group) were enrolled in the study.

**Fig. 1:**
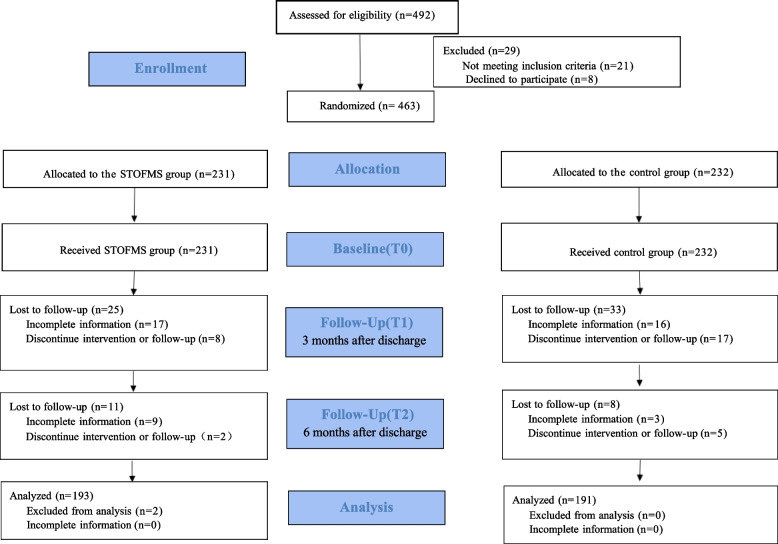
Flow diagram of the study

The demographic and medical characteristics of the participants are presented in Table 1. The Consolidated Standards for Reporting Trials reporting guidelines were followed (Fig. 1). In terms of demographic variables, the baseline data between the two groups were comparable at T0. After excluding the patients who lost to the final follow-up, the baseline data of the two groups were still comparable, without statistically significant difference. The primary and secondary outcomes are shown in Tables 2, 3 and 4.

**Table 1:** Characteristics all enrolled epileptic children and their families

Characteristics		STOFMS group (*n* = 231)	Control group (*n* = 232)	F/t	P
Child’s age, month, mean (SD)		47.13(42.18)	51.88(45.05)	1.171	0.242
Age of onset, month, mean (SD)		42.17(39.99)	46.85(42.47)	1.22	0.223
Child’s gender (%)	Male	112(0.49)	105(0.45)	0.484	0.487
	Female	119(0.52)	127(0.55)		
Parent’s age, year, mean (SD)		31.90(6.52)	31.51(6.33)	-0.656	0.512
Primary care giver’s gender (%)	Male	119(0.52)	132(0.57)	1.35	0.245
	Female	112(0.49)	100(0.43)		
Parental employment (%)	Employed	154(0.67)	167(0.72)	1.538	0.215
	Unemployed	77(0.33)	65(0.28)		
Parental education (%)	Below a Bachelor’s degree	201(0.87)	192(0.83)	1.633	0.201
	Bachelor’s degree and higher	30(0.13)	40(0.17)		
Time since diagnosis	≤ 3 months	173(0.75)	171(0.74)	1.525	0.822
	> 3–6 months	18(0.08)	24(0.1)		0.822
	0.5–1 year	11(0.05)	12(0.05)		0.822
	1–2 years	15(0.07)	11(0.05)		0.822
	> 2 years	14(0.06)	14(0.06)		0.822
Duration of medication (%)	None	46(0.2)	43(0.19)	1.733	0.785
	≤ 3 months	107(0.46)	112(0.48)		
	> 3–6 months	41(0.18)	48(0.21)		
	0.5–1 year	23(0.1)	18(0.08)		
	1–2 years	14(0.06)	11(0.05)		
Seizure frequency in the last 3 months		3.55(1.65)	3.49(1.51)	-0.427	0.669
Seizure duration	< 30 s	21(0.09)	18(0.08)	0.415	0.937
	31–59 s	86(0.37)	85(0.37)		
	1–3 min	80(0.35)	81(0.35)		
	3–5 min	44(0.19)	48(0.21)		
Frequency of hospitalization in the past month (%)	3 times or less	210(0.91)	217(0.94)	1.113	0.292
	More than 3 times	21(0.09)	15(0.07)		0.292
Payment (%)	Self-financed	36(0.16)	27(0.12)	1.534	0.216
	By health insurance	195 (0.84)	205(0.88)		
Annual income, CNY (%)	Less than 3000	38(0.17)	26(0.11)	2.952	0.229
	3000 to 5000	96(0.42)	97(0.42)		
	More than 5000	97(0.42)	109(0.47)

**Table 2:** Medication adherence and family management ability of two groups at different time points

Subscales (the maximum score)	Group	T0	T1	T2	*F*_*time*_	*P*	*F*_*group*_	*P*	*F*_*Interaction*_	*P*
Child's Daily Life (25 scores)	STOFMS	16.026(3.120)	19.005(3.314)	19.979(3.18)	99.896	< 0.001	42.582	< 0.001	19.199	< 0.001
	Control	16.01(3.332)	17.424(3.657)	17.529(2.914)						
	*t*	-0.047	-4.440	-7.873						
	*P*	0.963	< 0.001	< 0.001						
Condition Management Ability (60 scores)	STOFMS	33.72(4.745)	37.99(4.449)	38.995(3.314)	99.896	< 0.001	42.582	< 0.001	19.199	< 0.001
	Control	33.482(4.694)	37.016(4.84)	37.099(3.116)						
	*t*	-0.495	-2.053	-5.772						
	*P*	0.621	0.041	< 0.001						
Condition Management Effort (50 scores)	STOFMS	36.358(4.385)	36.067(1.195)	35.964(0.838)	0.880	0.416	1.089	0.297	0.050	0.951
	Control	36.492(4.418)	36.34(3.044)	36.251(3.606)						
	*t*	0.300	1.154	1.074						
	*P*	0.765	0.250	0.284						
Family Life Difficulty (70 scores)	STOFMS	47.959(4.271)	40.938(2.491)	39.829(3.741)	251.066	< 0.001	6.971	0.009	3.893	0.021
	Control	47.445(6.453)	41.995(2.885)	41.105(2.346)						
	*t*	-0.919	3.841	4.007						
	*P*	0.359	< 0.00114385505892902	< 0.0010762814109760762						
View of Condition Impact (20 scores)	STOFMS	13.995(2.408)	9.995(1.943)	9.026(1.718)	290.682	< 0.001	164.450	< 0.001	45.953	< 0.001
	Control	14.005(2.537)	12(1.947)	11.984(2.409)						
	*t*	0.041	10.101	13.840						
	*P*	0.967	< 0.001	< 0.001						
Parental Mutuality (40 scores)	STOFMS	30.995(4.114)	37.202(2.249)	38.383(2.094)	326.623	< 0.001	36.989	< 0.001	9.960	< 0.001
	Control	30.937(4.215)	36.021(3.005)	36.141(3.183)						
	*t*	-0.136	-4.357	-8.146						
	*P*	0.892	< 0.001	< 0.001

**Table 3:** Mean value and standard deviation (SD) at each time-point and overall between group comparison for 8-MMAS among two groups at different time points

Levels	T0			T1			T2			*F*_*time*_	*P*	*F*_*group*_	*P*	*F*_*Interaction*_	*P*
	STOFMS	Control	P	STOFMS	Control	P	STOFMS	Control	P						
Low compliance	155(0.8)	159(0.83)	0.456	9(0.05)	32(0.17)	< 0.001	0(0)	41(0.22)	< 0.001	558.092	< 0.001	49.325	< 0.001	102.239	< 0.001
Medium compliance	38(0.2)	32(0.17)		169(0.88)	152(0.8)		118(0.61)	147(0.77)							
High compliance	0(0.00)	0(0.00)		15(0.08)	7(0.04)		75(0.39)	3(0.02)							

Note: F_time_, F_group_, F_Interaction_ mean comparing the interaction actions between two groups at the three time points

**Table 4:** Clinical efficacy of two groups at different time points

	T1 Clinical efficacy			T1 Total efficacy		T2 Clinical efficacy			T2 Total efficacy	
	Markedly effective	Effective	Ineffective	Total Effective	Ineffective	Markedly effective	Effective	Ineffective	Total Effective	Ineffective
STOFMS	143(0.74)	33(0.17)	17(0.09)	176(0.91)	17(0.09)	144(0.75)	26(0.14)	23(0.12)	170(0.88)	23(0.12)
Control	108(0.57)	60(0.31)	23(0.12)	168(0.88)	23(0.12)	116(0.61)	37(0.19)	38(0.20)	153(0.80)	38(0.20)
*x2*	13.609			1.076		8.614			4.573	
P	0.001			0.300		0.013			0.032	

Note: Markedly effective: EEG returned to normal after treatment, clinical symptoms were completely relieved, and the frequency of seizures was reduced by more than 70%

Effective: EEG was generally normal, clinical symptoms were significantly improved, and the frequency of seizures was reduced by 50%

Ineffective: EEG and clinical symptoms were not significantly improved after treatment, and the frequency of seizures was reduced by 50% or less or there was no change

The total effective cases were markedly effective cases + effective cases

Figure 1 illustrates the complete RCT process. There were no significant differences in the basic demographics between the two groups (T0, Table 1). The parents’ ages were 31.90 ± 6.52 and 31.51 ± 6.33 in the STOFMS and control groups respectively. Most parents did not have a bachelor’s degree, and the monthly income of both groups were over RMB 5,000 (42.0% and 47.0% respectively).

### Analysis of ability of family management

The total scores and each subscale score on FaMM in the control and STOFMS groups at T0 exhibited no statistical significance (*p* > 0.05). Scores on FaMM collected at T1 increased in Child's Daily Life, Condition Management Ability, and Parental Mutuality while decreased in Condition Management Effort, Family Life Difficulty, and View of Condition Impact in both groups (*p* > 0.05). At T2, the positive scoring decreased in the control group, and slightly increased in the STOFMS group (*p* < 0.05), while the negative scoring increased in the control group, and slightly decreased in the STOFMS group (*p* < 0.05).

### Analysis of medication adherence and clinical efficacy

Tables 3 and 4 show the differences between the two groups at three time points. At the baseline, there were no significant differences in medication adherence and clinical efficacy. At T1, medication adherence improved in both groups, with a substantial higher increase in the STOFMS group compared to the control group (*p* < 0.001). At T2, medication adherence of the STOFMS group increased slightly with the number of children with high adherence increased, whereas that with low adherence decreased. However, medication adherence of the control group declined slightly, with less children with high adherence, and more children with low adherence, presenting a statistically significant difference after statistical analysis (*p* < 0.05). Table 4 shows that 3 months after discharge, there was no statistically significant difference in the clinical efficacy between the two groups. However, a substantial difference in the overall clinical outcomes was noted between these two groups 6 months after discharge. The total effective rate dropped at this point of time, yet children in the STOFMS group showed an 88.1% total effective rate, higher than that in the control group.

## Discussion

STOFMS is a developed family management intervention that incorporates a simulation connection based on family management education. It aims at improving the family management level of children and their parents, adherence with epilepsy treatment, and clinical efficacy. It provides important benefits to families of children with epilepsy when compared with the traditional model, as shown in the findings of this study. Besides, the previous conclusion that repeated seizures in children with epilepsy were commonly attributed to low medication adherence and caregivers’ poor disease awareness was also proved in our study [25]. This was probably owing to the limited self-management abilities of children with epilepsy and their parents’ insufficient management skills or disease-related knowledge, consistent with the socioeconomic and condition-related factors proposed by the WHO.

### The Ability of family management improved at 6 months after discharge

Considering the study’s findings, STOFMS was effective in improving management capacity, parental relationships and internal motivations. This may be attributed to the fact that families of children with epilepsy in China are often unable to cope with this condition due to a lack of understanding and excessive burden placed on family caregivers [26], as influenced by traditional Chinese culture that parents focus too much on the negative effects of the disease and have high expectations of medical care [27, 28]. STOFMS intervention is intended to build parents’ capacities to manage the epileptic disorder, rather than merely sharing disease-related knowledge. The whole-course management intervention from the child’s admission to 6 months after discharge was designed to educate parents about the importance of continued standard treatment and correct disease management behaviors [17]. Additionally, the researchers’ findings supported the argument that sufficient health education was associated with a family’s ability to correctly recognize and manage their children’s medical conditions [29].

### Medication adherence improved significantly in STOFMS group

The researchers proved for the first time that STOFMS was effective in promoting medication adherence in families of children with epilepsy. The study’s findings may provide useful management and educational interventions for these families. Specifically, caregivers were taught on the prevention and management of epilepsy, as well as the use of anti-seizure medicine. This protocol provides a new perspective on pediatric medication adherence interventions [30, 31], as the knowledge of epilepsy and disease treatment shared in the intervention has raised both the children and their parents’ awareness of regulatory use of anti-seizure medicine, thus changing their attitudes and medication behaviors. Furthermore, the family management level has been improved, resulting in better illness management and medication adherence. The results observed at 6 months after discharge suggested that maintaining stable outcomes of medication adherence in children with epilepsy at home required intensive long-term support. Predictably, without these ongoing connections between medical staff and patients, patients will demonstrate poorer medication adherence behavior. In this study, the follow-up system was used to remind children and their parents to take medicine, which improved children’s medication adherence, and regular visits to the hospital. The system also regularly pushed health education materials and emergency operation videos according to childrens’ disease progression and medication situation, thus improving their clinical efficacy as the families’ knowledge and emergency management ability strengthened. Also, the researchers findings confirmed the observation that patients’ medication adherence became more stable with the increase of intervention time [30, 31].

### Clinical symptom severity decreased significantly in STOFMS group

This study revealed that STOFMS could greatly improve the clinical efficacy of children with epilepsy. As shown in Table 4, the rate of clinical curative effect of the STOFMS group 3 months after discharge was slightly higher than that of the control group, but the improvement was not statistically significant (*p* > 0.05), given that anti-seizure medication therapy is a long-term process, and 3 months cannot produce the optimum results. Table 4 also showed the clinical efficacy of the STOFMS group was better than that of the control group 6 months after discharge. This was because standardized medication was proved to reliably enhance children’s clinical outcomes, and children, along with their parents, received guidance on taking effective measures based on the patient’s situation as family management strengthened. The STOFMS group hence knew how to reduce the inducing factors of epileptic seizures and identify early warning signals, enabling them to better manage children's disorders and increase clinical efficacy. In summary, the family management model raised family management level of children with epilepsy and further standardized their medication behavior and adherence, and therefore improved the clinical efficacy.

### Limitations

Despite the noteworthy findings, the study is subject to several limitations. First, there is response bias as the surveyed children and their parents may exaggerate their answers to the questionnaire or be unwilling to disclose their private information. A second limitation is that the impact of STOFMS on the enrolled children with different types of seizure has not been thoroughly investigated. It is suggested that future studies should group participants by types of seizure to further validate the application effect of STOFMS in this population. It is also important to highlight that the STOFMS group receive only a short-term intervention, so the efficacy of long-term intervention is yet to be examined, and its strategies should refer to the short-term intervention effectiveness in this study. Lastly, this is a single-centered study in which the researchers only include participants admitted to the researchers’ hospital, which may lead to selection bias. It is clear that further carefully designed research is needed to verify the effectiveness and feasibility of STOFMS.

## Conclusion

In light of the results of this study, STOFMS can improve the family management level, medication adherence, and clinical efficacy of children with epilepsy, which ultimately reduce the burden of epilepsy on children and their parents.

## Data Availability

Data are available from the corresponding author on reasonable requested.

## Abbreviations

STOFMS: Simulation Training Of Family Management Style
FaMM: Family Management Measure
FMSF: Family Management Style Framework
RCT: Randomized Controlled Trial
EEG: Electroencephalogram
MMAS-8: 8-item Morisky Medication Adherence Scale

## References

[1] Chang BL, Chang KH (2022). Stem Cell Therapy in Treating Epilepsy. Front Neurosci.

[2] Gea S (2020). Sander JW : The global burden of epilepsy report: Implications for low- and middle-income countries. Epilepsy Behav.

[3] Beghi E, Giussani G, Nichols E (2019). Global, regional, and national burden of epilepsy, 1990–2016: a systematic analysis for the Global Burden of Disease Study 2016. Lancet Neurol.

[4] Song P, Liu Y, Yu X, Wu J, Poon AN, Demaio A, Wang W, Rudan I, Chan KY (2017). Prevalence of epilepsy in China between 1990 and 2015: A systematic review and meta-analysis. J Glob Health.

[5] Li S, Wu J, Wang W, Jacoby A, de Boer H, Sander JW (2010). Stigma and epilepsy: the Chinese perspective. Epilepsy & behavior : E&B.

[6] Romeo DM, Venezia I, Pede E, Brogna C (2023). Cerebral palsy and sex differences in children: A narrative review of the literature. J Neurosci Res.

[7] Holmes GL (2023). Commentary on the Paper "Effect of Seizures on the Developing Brain and Cognition". Seminars in pediatric neurology.

[8] Operto FF, Pastorino GMG, Viggiano A, Dell'Isola GB, Dini G, Verrotti A, Coppola G (2023). Epilepsy and Cognitive Impairment in Childhood and Adolescence: A Mini-Review. Curr Neuropharmacol.

[9] Perucca P, Scheffer IE, Kiley M (2018). The management of epilepsy in children and adults. Med J Aust.

[10] Makridis KL, Bast T, Prager C, Kovacevic-Preradovic T, Bittigau P, Mayer T, Breuer E, Kaindl AM (2022). Real-World Experience Treating Pediatric Epilepsy Patients With Cenobamate. Front Neurol.

[11] Modi AC, Guilfoyle SM, Glauser TA, Mara CA (2021). Supporting treatment adherence regimens in children with epilepsy: A randomized clinical trial. Epilepsia.

[12] Dima SA, Shibeshi MS (2022). Antiepileptic drug adherence in children in southern Ethiopia: A cross sectional study. PLoS ONE.

[13] Beacham BL, Deatrick JA (2019). Adapting the Family Management Styles Framework to Include Children. J Pediatr Nurs.

[14] Ma J, Yu Q, Zhang T, Zhang Y (2020). Chinese family care patterns of childhood rheumatic diseases: A cluster analysis. Int J Nurs Sci.

[15] Al GHN, Winter MA, Everhart RS (2017). Examining Profiles of Family Functioning in Pediatric Asthma: Longitudinal Associations With Child Adjustment and Asthma Severity. J Pediatr Psychol.

[16] Lima-Rodríguez JS, Lima-Serrano M, Domínguez-Sánchez I (2015). Psychometric properties of an instrument to measure family disease management. Int J Clin Health Psychol.

[17] Im Y, Cho Y, Kim D (2019). Family Management Style as a Mediator between Parenting Stress and Quality of Life of Children with Epilepsy. J Pediatr Nurs.

[18] Liu H, Song Q, Zhu L, Chen D, Xie J, Hu S, Zeng S, Tan L (2020). Family Management Style Improves Family Quality of Life in Children With Epilepsy: A Randomized Controlled Trial. J Neurosci Nurs.

[19] Liu H (2017). GU L, Zhu L, LI Y, Chen D, Shu T, Zhang Q: Effect of family management intervention in children with epilepsy. Chin J Nurs.

[20] Knafl K, Deatrick JA, Gallo A, Dixon J, Grey M, Knafl G, O’Malley J (2011). Assessment of the Psychometric Properties of the Family Management Measure. J Pediatr Psychol.

[21] Schulz KF, Altman DG, Moher D (2010). CONSORT 2010 statement: updated guidelines for reporting parallel group randomised trials. PLoS Med.

[22] Zhang Y (2009). Study on family management mode and family intervention effect of children with chronic diseases.

[23] Islam T, Muntner P, Webber LS, Morisky DE, Krousel-Wood MA (2008). Cohort study of medication adherence in older adults (CoSMO): extended effects of Hurricane Katrina on medication adherence among older adults. Am J Med Sci.

[24] Li X (2021). Application of family management model in continuing care of children with epilepsy.

[25] Tolchin B, Dworetzky BA, Baslet G (2018). Long-term adherence with psychiatric treatment among patients with psychogenic nonepileptic seizures. Epilepsia.

[26] Paschal AM, Mitchell QP, Wilroy JD, Hawley SR, Mitchell JB (2016). Parent health literacy and adherence-related outcomes in children with epilepsy. Epilepsy & behavior : E&B.

[27] Yang C, Yao T, Huang Y, Zhao L, Zhang L (2021). Prevalence and influencing factors of depression of caregivers in children with epilepsy in southwestern China: a cross-sectional study. Medicine.

[28] Yang C, Kang B, Mao Y, Xu Q, Yu D, Zhang L (2020). Anxiety among caregivers of children with epilepsy from western China: A cross-sectional survey. Medicine.

[29] Zaidman EA, Scott KM, Hahn D, Bennett P, Caldwell PH (2023). Impact of parental health literacy on the health outcomes of children with chronic disease globally: A systematic review. J Paediatr Child Health.

[30] Brashers DE, Basinger ED, Rintamaki LS, Caughlin JP, Para M (2017). Taking Control: The Efficacy and Durability of a Peer-Led Uncertainty Management Intervention for People Recently Diagnosed With HIV. Health Commun.

[31] Sheibani R, Sheibani M, Heidari-Bakavoli A, Abu-Hanna A, Eslami S (2017). The Effect of a Clinical Decision Support System on Improving Adherence to Guideline in the Treatment of Atrial Fibrillation: An Interrupted Time Series Study. J Med Syst.

